# Gut dysbiosis induced by cardiac pressure overload enhances adverse cardiac remodeling in a T cell-dependent manner

**DOI:** 10.1080/19490976.2020.1823801

**Published:** 2020-10-25

**Authors:** Francisco J Carrillo-Salinas, Marina Anastasiou, Njabulo Ngwenyama, Kuljeet Kaur, Albert Tai, Sasha A. Smolgovsky, David Jetton, Mark Aronovitz, Pilar Alcaide

**Affiliations:** a Department of Immunology, Tufts University School of Medicine, Boston, MA, USA; b Department of Internal Medicine, University of Crete Medical School, Crete, Greece; c Department of Immunology, Tufts Graduate School for Biomedical Sciences Immunology Program, Tufts University School of Medicine, Boston, MA, USA

**Keywords:** Heart failure, t cells, gut microbiota, aryl hydrocarbon receptor, inflammation

## Abstract

Despite the existing association of gut dysbiosis and T cell inflammation in heart failure (HF), whether and how gut microbes contribute to T cell immune responses, cardiac fibrosis and dysfunction in HF remains largely unexplored. Our objective was to investigate whether gut dysbiosis is induced by cardiac pressure overload, and its effect in T cell activation, adverse cardiac remodeling, and cardiac dysfunction.

We used 16S rRNA sequencing of fecal samples and discovered that cardiac pressure overload-induced by transverse aortic constriction (TAC) results in gut dysbiosis, characterized by a reduction of tryptophan and short-chain fatty acids producing bacteria in WT mice, but not in T cell-deficient mice (*Tcra^−/-^*) mice. These changes did not result in T cell activation in the gut or gut barrier disruption. Strikingly, microbiota depletion in WT mice resulted in decreased heart T cell infiltration, decreased cardiac fibrosis, and protection from systolic dysfunction in response to TAC. Spontaneous reconstitution of the microbiota partially reversed these effects. We observed decreased cardiac expression of the Aryl hydrocarbon receptor (AhR) and enzymes associated with tryptophan metabolism in WT mice, but not in *Tcra^−/-^* mice, or in mice depleted of the microbiota.

These findings demonstrate that cardiac pressure overload induced gut dysbiosis and T cell immune responses contribute to adverse cardiac remodeling, and identify the potential contribution of tryptophan metabolites and the AhR to protection from adverse cardiac remodeling and systolic dysfunction in HF.

## Graphical abstract

**Figure:**
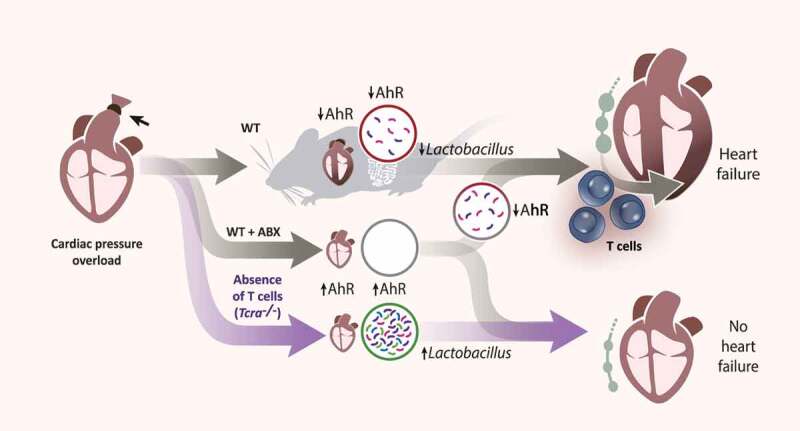

## INTRODUCTION

Alterations in the gut microbiota, a process known as gut dysbiosis,^1^ correlate with the severity of the complex syndrome of heart failure (HF), a leading cause of mortality affecting more than 24 million people worldwide.^2^ The current paradigm is that intestinal edema and congestion observed in chronic HF patients contribute to increased gut permeability, lipopolysaccharide (LPS) leakage and inflammation,^3–6^ characterized in part, by a high frequency of circulating pro-inflammatory T cells.^7^

The gut is one of the main reservoirs of T cells in the body, which are in close contact with gut bacteria to maintain homeostatic control of local and systemic immunity. This is often mediated by metabolites processed by the gut microbiota.^8–10^ These metabolites include short-chain fatty acids (SCFAs) and tryptophan derivatives, which are further metabolized by the mammalian enzymes indoleamine 2, 3-dioxygenase (IDO1), and kynurenine formamidase (AFMID), and activate the SCFA receptors and aryl hydrocarbon receptor (AhR), respectively, in host cells, and modulate homeostatic immune responses. Disruption of this process can lead to over-activation of T cells in the gut lamina propria and influence immune responses in distant sites during chronic inflammatory responses.^11^

Numerous gut microbial metabolic pathways, including the production of trimethylamine N-oxide (TMAO) and secondary bile acids, have been reported to contribute to the progression of several cardiovascular diseases, including HF.^1^ Studies in mice subjected to cardiac pressure overload induced by transverse aortic constriction (TAC), a well-established non-ischemic HF model, evidenced that TMAO supplementation worsened adverse cardiac remodeling and cardiac dysfunction.^12^ More recently, TMAO was shown to induce myocardial fibrosis and cardiac hypertrophy in rats subjected to TAC.^13^ The direct role of the microbiota in cardiovascular disease outcomes has also recently been studied in germ-free mice or in antibiotic (ABX) treated mice. While microbiota depletion in mice has been shown to be harmful during myocardial infarction (MI),^14^ it protects from autoimmune myocarditis^15^ and was reported to be beneficial in angiotensin-II induced hypertension.^16^ This somewhat parallels observations in T cell-deficient mice demonstrating that while T cells are required for myocardial healing after MI,^17^ they are detrimental in inflammation associated with hypertension.^18^ These results suggest the intriguing possibility that microbiota may go along with T cell activation in cardiac inflammation. We and others have demonstrated that TAC induces T cell activation in the mediastinal lymph nodes (LNs) that drain the heart, as well as heart T cell infiltration.^19,20^ We further demonstrated that heart infiltrated IFNγ-producing T cells induced cardiac fibrosis and dysfunction.^21,22^ However, whether TAC induces gut dysbiosis that modulates T cell immune responses and adverse cardiac remodeling, and the outcome of depleting the gut microbiota in the onset of TAC remains unknown.

Here, we report the novel finding that microbiota depletion at the onset of TAC dampens T cell activation and prevents adverse cardiac remodeling and cardiac dysfunction. We identify distinct bacterial populations and metabolic pathways induced by TAC in wild type (WT) and *Tcra^−/-^* mice, and a protective mechanism that involves a tryptophan/AhR axis.

## RESULTS

### *Cardiac pressure overload induced by TAC results in gut microbiota alterations in WT but not in T cell-deficient mice (*Tcra*^−/-^)*

**Figure 1.**
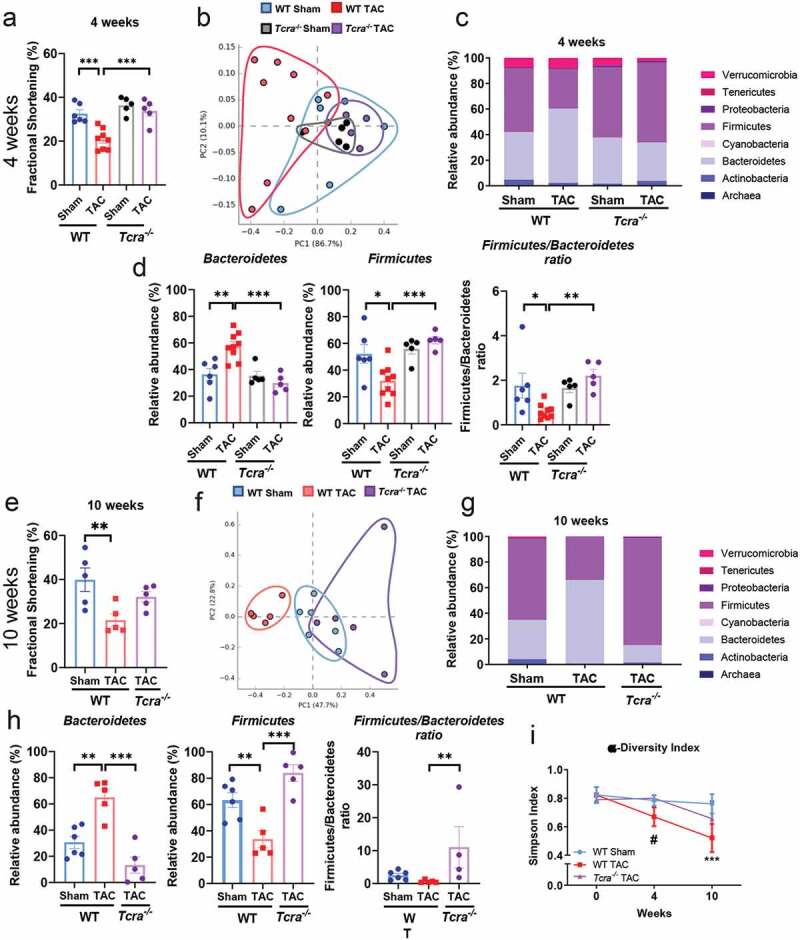
**Cardiac pressure overload induces gut dysbiosis characterized by a low Firmicutes/Bacteroidetes ratio in WT but not in *Tcra^−/-^* mice**. Wild type (WT) and *Tcra*^−/-^ mice were subjected to 4 weeks (a-d, i) or 10 weeks (e-i) Sham or TAC surgery and echocardiography was performed at 4 weeks (a) and 10 weeks post-surgery (e). Principal component analysis (PCA) clustering of gut microbial populations generated after 16S rRNA sequencing of fecal pellets of WT and *Tcra*^−/-^ at 4 weeks (b) and 10 weeks (f) post-Sham or TAC surgery. Each point represents one mouse. Relative abundance of main phyla of gut microbiota in fecal pellets (c, g), and specifically Bacteroidetes, Firmicutes and Firmicutes/Bacteroidetes ratio (d, h), from WT and *Tcra*^−/-^ mice harvested at 4 weeks (c, d) and 10 weeks post-Sham or TAC (g, h). Alpha diversity representation over time in WT and *Tcra*^−/-^ mice (i). *p < .05; **p < .01; ***p < .001; ns = non statistically significant. Statistical analysis: ANOVA test with a Tukey-Kramer posthoc test (a-j), and 2-way ANOVA test and Tukey’s multiple comparison test were used (**I**). #p < .05 WT TAC vs *Tcra^−/-^* TAC; ***p < .001 WT Sham vs WT TAC (**I**)

We evaluated systolic function by echocardiography and performed 16S rRNA sequencing of fecal samples from mice subjected to TAC or Sham surgery at 4 and 10 weeks post-TAC, in order to assess if TAC-induced dysbiosis was associated with systolic dysfunction. In addition, we evaluated the role of T cells in the microbiota composition in response to TAC in *Tcra^−/-^* mice. As we previously reported,^19^ *Tcra^−/-^* showed increased percentage of fractional shortening as compared to WT mice in response to TAC at 4 weeks, and comparable to WT and *Tcra^−/-^* Sham mice (Figure 1a) and 10 weeks post-surgery (Figure 1e), confirming a role of T cells in systolic dysfunction. The gut microbiota profiles in WT and *Tcra^−/-^* was very similar at baseline (Online Figure 1a) with no significant changes in phyla (Supplemental Figure 1b) and only minor variations in genera relative abundance (Supplemental Figure 1c). Furthermore, the microbiota profile of WT and *Tcra^−/-^* control mice did not significantly change with age between 8 and 18 weeks of age in WT and 8 to 12 weeks of age in *Tcra^−/-^* mice (Supplemental Figure 1d, 1e).

We found that TAC-induced gut microbiota alterations in WT mice, compared to Sham at 4 weeks post-surgery, as indicated in the principal component analysis (PCA; Figure 1b). We also analyzed the relative abundances of bacterial communities assigned to each phylum. Phylum Bacteroidetes was enriched after 4 weeks of cardiac pressure overload (Figure 1c, 1d), whereas the phylum Firmicutes was reduced in WT TAC mice as compared to Sham (Figure 1c, 1d). A lower Firmicutes/Bacteroidetes ratio, associated with inflammation, was observed in WT TAC mice compared to WT Sham mice (Figure 1d). Strikingly, the gut microbiota alterations observed in WT TAC mice were not evident in *Tcra^−/-^* TAC mice, which resembled to WT Sham and *Tcra^−/-^* Sham, and significantly differed from WT TAC mice at 4 weeks (Figure 1b-1d).

At 10 weeks post-TAC, which mimics a later stage of HF and is associated with gut dysbiosis in patients,^1^ WT TAC mice showed sustained systolic dysfunction (Figure 1e), and differences between WT Sham and TAC gut microbiota profiles were more evident (Figure 1f-1h). A significant increase of phylum Bacteroidetes and a decrease in phylum Firmicutes were still observed in WT TAC mice as compared to Sham mice (Figure 1g, 1h). The similarity in bacterial phyla composition between WT Sham and *Tcra^−/-^* TAC was also sustained at 10 weeks post-TAC (Figure 1f-1h), when *Tcra^−/-^* percentage of fractional shortening was comparable to Sham WT mice (Figure 1e). The alpha diversity, determined by the Simpson index, was also decreased in WT mice over time in response to TAC, in contrast to *Tcra^−/-^* mice (Figure 1i).

Taken together, these results indicate that cardiac pressure overload induces gut dysbiosis in WT mice as they develop systolic dysfunction overtime, and demonstrate the requirement of T cells for TAC-induced gut dysbiosis. Our results further identify the induction of Bacteroidetes and reduction of Firmicutes phyla by cardiac pressure overload in WT but not in *Tcra^−/-^* mice.

### TAC-induced gut dysbiosis does not result in T cell activation in the lamina propria or in gut epithelial barrier disruption

**Figure 2.**
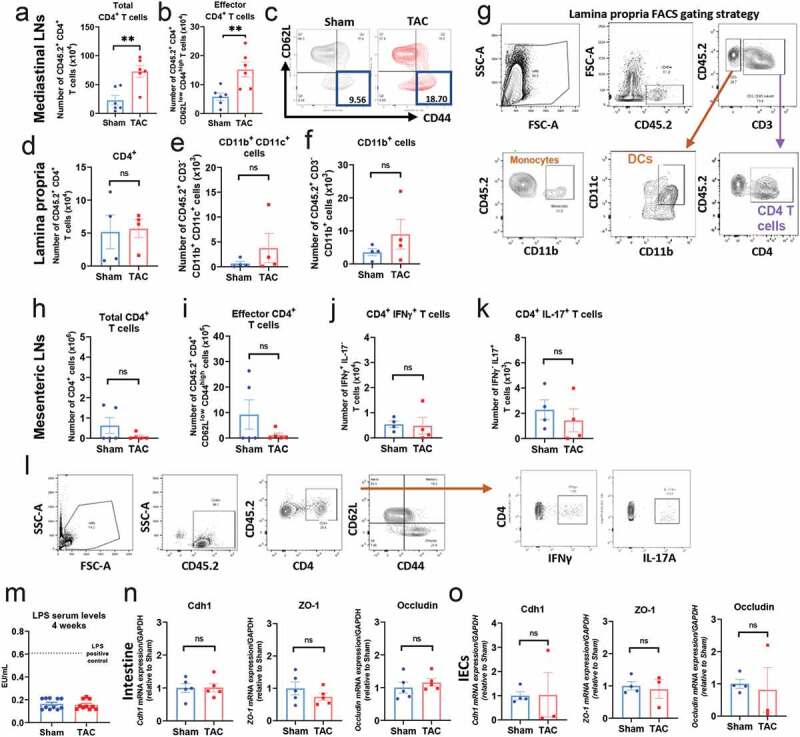
**Cardiac pressure overload results in increased numbers of effector T cells in the mediastinal LNs, and does not induce T cell activation in the lamina propria, or in expression of epithelial markers of gut barrier disruption.** a, b, Flow cytometry quantification of total number of CD4^+^ T cells (a), and quantification and representative plots (b, c) of CD4^+^ CD62L^low^ CD44^hi^ T cells in the mediastinal LNs of 4 weeks post-Sham and TAC. d-g, Flow cytometry quantification of CD4^+^ T cells (d), CD11b^+^CD11c^+^ cells (DCs, e), and CD11b^+^ (monocytes, f), in the lamina propria of 4 weeks post-Sham and TAC mice. (g), representative plots for lamina propria FACS gating strategy. (h-l), flow cytometry quantification of number of CD4^+^ T cells (h), CD62L^low^ CD44^high^ effector T cells (i), IFNγ^+^ T cells (j) and IL-17^+^ T cells (k) in the mesenteric LNs, the draining LNs of the gut, and flow cytometry gating strategy (l). (m), Quantification of LPS concentration by ELISA in the serum of WT mice 4 weeks post-Sham and TAC. (n-o), Gene expression levels of Cdh1, ZO-1 and occludin in the gut (n) and in isolated intestinal epithelial cells (IECs) (o) from WT Sham and TAC mice 4 weeks post-TAC. **p < .01; Student t-test was used for statistical analysis

Communication between gut resident T cells and the microbiota is critical for intestinal homeostasis. Based on our findings that TAC-induced changes in the microbiota, we hypothesized that gut dysbiosis induced by TAC resulted in T cell activation in the gut and possible disruption of barrier integrity. As expected, TAC triggered an increase in the numbers of T cells (Figure 2a) and activated effector CD4^+^ CD62L^low^ CD44 ^high^ T cells in the mediastinal LNs (Figure 2b, 2c). Interestingly, despite the observed gut microbiota alterations induced by TAC, the numbers of immune cells in the lamina propria, which are critical to maintain homeostatic T cell immune responses in the gut, such as T cells themselves (Figure 2d), dendritic cells (DCs, Figure 2e) and monocytes (Figure 2f), were similar between Sham and TAC mice. We further analyzed the gut draining LNs and found similar numbers of T cells (Figure 2h), activated effector CD4^+^ CD62L^low^ CD44^high^ T cells (Figure 2i), effector T helper (Th) type 1 (Figure 2j), and Th type 17 (Figure 2k) cells in the mesenteric LNs of Sham and TAC mice.

Chronic HF patients usually develop intestinal edema and congestion that may contribute to increased gut permeability and LPS leakage, and is hypothetically associated with chronic inflammation.^6,23^ In this context, we next investigated whether TAC-induced gut barrier disruption, by measuring LPS levels in serum (as a read out of bacterial leakage), and the expression of several epithelial cell junctional genes, such as *zonula occludens*-1 (ZO-1), cadherin-1 (Cdh-1), or occludin (Figure 2m-2o). The concentration of serum LPS was similar between Sham and TAC mice, and significantly decreased as compared to mice injected with LPS as a positive control (Figure 2m). These results were in line with the similar expression of ZO-1, Cdh-1 and occludin in the intestine (Figure 2n) or specifically in intestinal epithelial cells (IECs) isolated from the intestines of Sham and TAC mice (Figure 2o). Taken together, these data demonstrate that TAC-induced gut dysbiosis does not result in T cell activation in the lamina propria and the mesenteric LNs, or in changes in Th1 and Th17 effector T cell populations. Moreover, TAC does not alter the gene expression of epithelial cell junctional molecules.

### Gut microbiota depletion preserves cardiac function and prevents adverse cardiac remodeling induced by TAC

**Figure 3.**
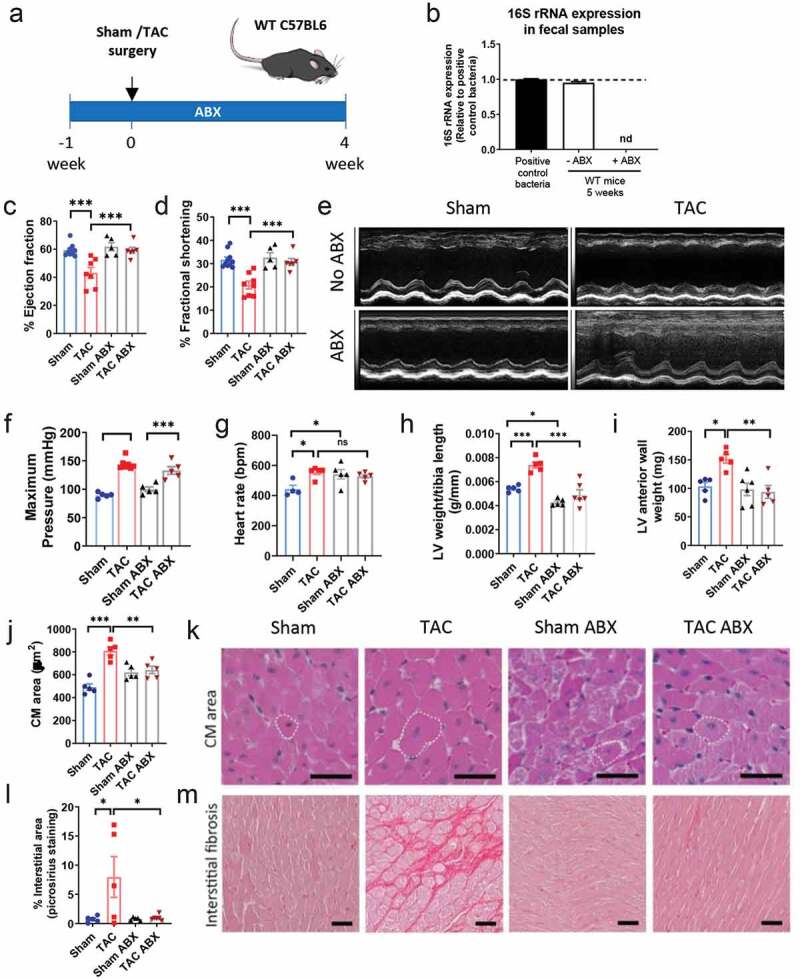
Gut microbiota depletion preserves systolic function in response to TAC and prevents adverse cardiac remodeling

To address the direct impact of the gut microbiota in the development of cardiac dysfunction induced by cardiac pressure overload, we depleted the gut microbiota in WT mice with ABX treatment and analyzed mice at a time in which mice develop adverse cardiac remodeling and cardiac dysfunction, as we have described (Figure 3a).^19^ qPCR of 16S rRNA expression in fecal samples demonstrated sterilization of the gut with ABX treatment at the onset of TAC (Figure 3b). TAC resulted in decreased ejection fraction (Figure 3c) as well as fractional shortening (Figure 3d) as compared to Sham (Table 2). Interestingly, the decline in percentage of ejection fraction and percentage of fractional shortening was not observed in the absence of the microbiota (TAC ABX mice) and was comparable between Sham and TAC ABX-treated mice (Figure 3c-3e), despite the expected similar increase in maximum pressure due to aortic constriction in TAC and TAC ABX mice (Figure 3f). As expected, the heart rate was increased in WT mice in response to TAC, and, surprisingly was also similarly increased in Sham ABX. However, TAC did not induce a further increase in heart rate in ABX treated mice (Figure 3g). The stroke volume trended toward being lower in those conditions with higher heart rate (TAC, Sham ABX, and TAC ABX), and these resulted in comparable cardiac outputs among the four groups (Table 2). We further studied several parameters to assess the presence of adverse cardiac remodeling in these groups. TAC ABX mice did not develop LV hypertrophy in response to TAC, evaluated as LV/tibia length ratio (Figure 3h) and LV anterior wall mass (measured by echocardiography; Figure 3i), compared to TAC group with gut microbiota.

**Table 1.** Primer sequences used in this study

Gene	Forward primer sequence	Reverse primer sequence
*AFMID*	5ʹ-TTG GGA ACT TCG TGC AGA TAG-3’	5ʹ-CAG TTT CTC CCC TTC GCC ATC −3’
*AhR*	5ʹ-ACC AGA ACT GTG AGG GTT GG-3’	5ʹ-TCT GAG GTG CCT GAA CTC CT-3’
*Cdh-1*	5ʹ-CAG CCT TCT TTT CGG AAG ACT-3´	5ʹ-GGT AGA CAG CTC CCT ATG ACT G-3’
*Cyp1a1*	5ʹ- CCT CAT GTA CCT GGT AAC CA-3’	5ʹ- AAG GAT GAA TGC CGG AAG GT-3’
*Cyp1b1*	5ʹ- CTG AGT TGG ACC AGG TTG TGG-3’	5ʹ- CAT GGA TTC TAA ACG ACT AGG-3’
*Cyp2e1*	5ʹ- ATG AGA GCC GGA TCC AGA G-3’	5ʹ- AGA GGA TGT CGG CTA TGA CG-3’
*FFR3 (GPR41)*	5ʹ-GTG ACC ATG GGG ACA AGC TTC-3’	5ʹ-CCC TGG CTG TAG GTT GCA TT-3’
*FFR2 (GPR43)*	5ʹ-GGC TTC TAC AGC AGC ATC TA-3’	5ʹ-AAG CAC ACC AGG AAA TTA AG-3’
*Ido1*	5ʹ-GCT TTG CTC TAC CAC ATC CAC −3’	5ʹ-CAG GCG CTG TAA CCT GTG T − 3’
*SLC16A1 (MCT-1)*	5ʹ-CAT TGG TGT TAT TGG AGG TC-3’	5ʹ-GAA AGC CTG ATT AAG TGG AG-3’
*Occludin*	5ʹ-ACT GGG TCA GGG AAT ATC CA-3’	5ʹ-TCA GCA GCA GCC ATG TAC TC-3’
*Socs1*	5ʹ-CTG CGG CTT CTA TTG GGG AC −3’	5ʹ-AAA AGG CAG TCG AAG GTC TCG −3’
*Socs3*	5ʹ-ATG GTC ACC CAC AGC AAG TTT −3’	5ʹ-TCC AGT AGA ATC CGC TCT CCT −3’
*ZO-1*	5ʹ-GGG AGG GTC AAA TGA AGA CA-3’	5ʹ-GGC ATT CCT GCT GGT TAC AT-3’
*16S*	5ʹ-AGA GTT TGA TCM TGG CTC AG-3’	5ʹ-AAG GAG GTG ATC CAN CCR CA-3’

**Table 2.** Characterization of cardiac function in the groups that were used in the study. Ejection fraction, fractional shortening, anterior wall mass, anterior wall thickness, and posterior wall thickness were measured by echocardiography. Heart rate, stroke volume, and cardiac output were determined by invasive hemodynamics. *p < .05, ***p < .001 Sham vs TAC; ^††^p < .01,^†††^p < .001 TAC vs TAC ABX or TAC REC, ANOVA test; ^§^p < .05 TAC ABX vs TAC REC, Student t-test. Values are means ± SEM

Parameters	Sham	TAC	Sham ABX	TAC ABX	TAC REC
Ejection fraction (%)	59.85 ± 2.62	40.96 ± 4.64***	61.81 ± 2.90	59.22 ± 2.14^†††^	47.98 ± 4.03^§^
Fractional shortening (%)	32.55 ± 1.77	19.64 ± 1.80***	32.63 ± 2.09	30.75 ± 1.47^†††^	24.01 ± 2.46^§^
Anterior wall mass (mg)	99.49 ± 6.33	160.34 ± 7.90*	91.76 ± 9.72	99.85 ± 11.68^††^	128.22 ± 10.29
Anterior wall thickness (mm)	1.41 ± 0.08	1.51 ± 0.06	1.18 ± 0.08	1.35 ± 0.10	1.39 ± 0.16
Posterior wall thickness (mm)	1.16 ± 0.10	1.26 ± 0.03	1.41 ± 0.14	1.27 ± 0.10	1.29 ± 0.04
Heart rate (bpm)	443.25 ± 25.24	551.8 ± 10.03	541.4 ± 31.74	527.4 ± 12.19	
Stroke volume (µl)	20.75 ± 1.79	16.6 ± 2.13	15.6 ± 2.82	12.8 ± 1.59	
Cardiac output (µl/min)	9180.25 ± 853.57	9147 ± 1153.21	8148 ± 1137.44	6764.6 ± 865.80

(a), Experimental design for gut microbiota depletion with antibiotics cocktail (ABX) during TAC in WT mice. (b), 16S rRNA expression measured by qPCR, from fecal pellets of WT mice provided with water or water supplemented with ABX treatment, 4 weeks post-Sham or TAC surgery. (c-e), Ejection fraction (c) and fractional shortening (d) were analyzed by echocardiography, and representative M-mode images are shown (e). Maximum pressure (f) and heart rate (g) were measured by invasive hemodynamics at the end of the experiment. Analysis of LV weight and tibia length ratio (h), LV anterior wall weight (i, determined by echocardiography), cardiomyocyte (CM) area (j), and interstitial fibrosis (l). k, representative hematoxylin/eosin (H&E) images of LV sections. m, representative pictures of LV sections with picrosirius red staining for collagen deposition. Dotted line: representative cardiomyocytes. Scale bar: 50 μm. Each dot represents one animal. ANOVA test with Tukey post hoc test; *p < .05; **p < .01; ***p < .001; nd: not determined; ns: not significant.

We next evaluated cardiomyocyte hypertrophy and found that the myocyte cross-sectional area was decreased in TAC ABX mice as compared to TAC mice (Figure 3j, 3k). Gut microbiota depletion also resulted in amelioration of LV interstitial fibrosis, another hallmark of adverse cardiac remodeling, in response to TAC (Figure 3l, 3m). Taken together, these data indicate that the gut microbiota present in TAC mice modulates adverse cardiac remodeling and systolic function, and evidences the existence of a gut-heart axis in the context of cardiac pressure overload.

**Gut microbiota depletion limits T cell activation in the mediastinal LNs and results in decreased cardiac LV T cell infiltration induced by TAC.**

**Figure 4.**
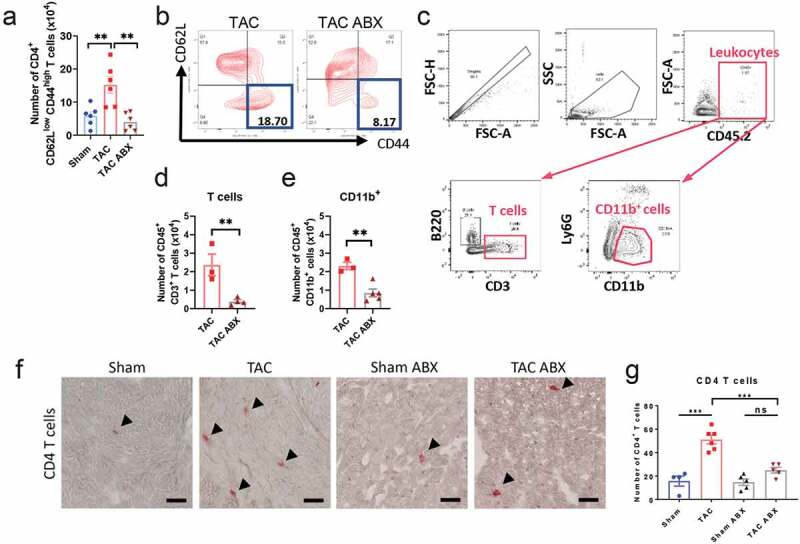
**Depletion of the gut microbiota results in reduced number of activated CD4^+^ T cells in the mediastinal LNs and reduced T cell heart infiltration**. WT mice were treated with ABX and subjected to 4 weeks TAC or Sham, as in Fig 3. Flow cytometry quantification (a) and representative plots (b) of CD4^+^ CD62L^low^ CD44^high^ effector T cells in the mediastinal LNs of Sham and TAC with or without ABX treatment. Hearts were digested and analyzed by flow cytometry using the indicated gating strategy (c). T cells (d) and CD11b^+^ cells (e) were quantified. Representative pictures of a CD4 immunostaining (**F**, arrows) and quantification of CD4^+^ T cells (g) in LV sections in Sham and TAC mice treated with or without ABX. Scale bar: 50 μm. ANOVA test (a, g) or Student t test (d, e) were used; **p < .01; ***p < .001; ns: not significant

As reported earlier, cardiac pressure overload induces specific T cell activation in the mediastinal LNs (Figure 2a, 2b), and infiltrated CD4 T cells are major contributors to adverse cardiac remodeling and cardiac fibrosis.^19–22^ In addition, the microbiota profile of *Tcra^−/-^* TAC mice is significantly different from WT TAC mice and more closely resembles Sham mice (Figure 1a-1c). Given the importance T cells have in the pathogenesis of TAC-induced adverse cardiac remodeling and systolic dysfunction, we analyzed T cell activation in the mediastinal LNs of TAC ABX mice. Gut microbiota depletion by ABX significantly decreased T cell activation during TAC in the mediastinal LNs (CD4^+^ CD62L^low^ CD44^high^ T cells; Figure 4a, 4b). We further assessed the number of T cells and monocytes in the digested LV of TAC and TAC ABX mice. Interestingly, CD4^+^ T cells were significantly reduced (Figure 4d) in the absence of gut microbiota during cardiac pressure overload. These results were corroborated by immunohistochemistry of LV sections, in which TAC mice showed a significant increase in the number of CD4 T cells in the LV, compared to Sham group (Figure 4f, 4g), and gut microbiota-depleted TAC mice evidenced significant less number of infiltrated CD4 T cells when compared to TAC mice (Figure 4f, 4g). The number of CD45^+^ CD11b^+^ monocytes was also reduced in TAC mice after gut microbiota removal (Figure 4e).

Collectively, these results indicate that gut sterilization results in decreased CD4^+^ T cell expansion in the mediastinal LNs, as well as in reduced CD4^+^ T cell activation and left ventricular infiltration, as compared to WT untreated mice at 4 weeks post-TAC. This reduction in CD4^+^ T cell infiltration may be responsible for preventing the development of adverse cardiac remodeling and systolic dysfunction in response to TAC in mice lacking microbiota.

**SCFA- and tryptophan-related metabolic pathways are differentially induced by cardiac pressure overload in WT and *Tcra^−/-^* mice.**

**Figure 5.**
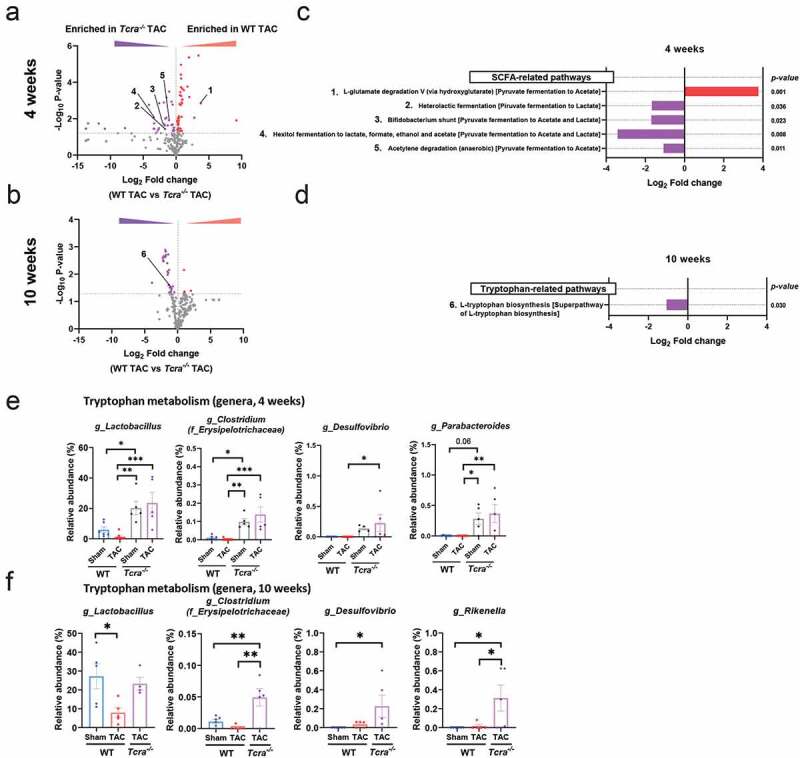
**Changes in SCFA and tryptophan associated metabolic pathways are induced by TAC over time, and dependent of T cell presence**. Volcano plots showing the degree of differential expression of bacterial metabolic pathways in WT and *Tcra*^−/-^ mice at 4 weeks (a) and 10 weeks (b) post-TAC. Specifically, numbered SCFA-enriched pathways and tryptophan-related pathways represented in A and B are described (c, d). Red: enriched pathways in WT TAC vs *Tcra^−/-^* TAC; Purple: enriched pathways in *Tcra^−/-^* TAC vs WT TAC gut microbiota. Relative abundances of genera which can metabolize tryptophan that significantly changed at 4 weeks post Sham or TAC in WT and *Tcra*^−/-^ mice (e) and 10 weeks (f) post-TAC are shown. For statistical analysis, we used Welch´s t test (a-d) and parametric ANOVA test (e, f). The dotted horizontal line in A and B represents the 0.05 *p*-value cutoff. *p < .05; **p < .01; ***p < .001; ns: not significant. g_(genus), f_(family)

Our data so far indicated that both microbes and T cells are central players of adverse cardiac remodeling induced by TAC. However, we did not observe any signs of leaky gut in TAC mice (Figure 2m-2o). We hypothesized that bacterial metabolites regulate adverse cardiac remodeling in response to TAC through T cell-dependent mechanisms. Thus, we focused on metabolic pathway analysis between WT vs *Tcra^−/-^* mice in response to TAC over time. The metabolic pathway abundance was determined by 16S rRNA sequencing in fecal samples followed by analysis with the *MetaCyc* database.^24^ Our analysis revealed that, among the predominantly enriched metabolic pathways in *Tcra^−/-^* mice vs WT mice at 4 and 10 weeks post TAC, SCFA- and tryptophan-related pathways, both of which have been associated with immunomodulation in cardiovascular disease^14,25^ stood out as differentially enriched in *Tcra^−/-^* mice. Specifically, SCFA-related pathways were enriched in *Tcra^−/-^* mice vs WT at 4 weeks post-TAC, whereas the tryptophan biosynthesis pathway was enriched at 10 weeks in *Tcra^−/-^* TAC compared to Sham (Figure 5a-5d). The only SCFA-related metabolic pathway enriched in WT TAC at 4 weeks was the L-glutamate degradation V (“Pyruvate fermentation to acetate” class) (Figure 5c), while the “pyruvate fermentation to acetate and/or lactate” pathways were enriched in *Tcra^−/-^* mice (Figure 5a, 5c). These pathways were associated with an increased relative abundance of the genus *Lactobacillus* in Sham and TAC *Tcra^−/-^* mice vs WT TAC. *Lactobacillus* plays a role in gut homeostasis in part by pyruvate fermentation to produce SCFA and metabolism of tryptophan,^26^ and was also significantly decreased in response to TAC in WT mice as compared to Sham mice (Figure 5e). Further taxonomic analysis demonstrated that other genera such as *Clostridium* and *Parabacteroides*, also shown to participate in SCFA and tryptophan metabolism, were significantly increased in Sham and TAC *Tcra^−/-^* mice compared to WT TAC, and genus *Desulfovibrio* was also found increased in *Tcra^−/-^* TAC mice vs WT TAC mice (Figure 5e), although these were overall relatively less represented than *Lactobacillus*. A similar relative abundance of all these bacterial genera was observed at 10 weeks post TAC (Figure 5f). Specifically, genus *Lactobacillus* represented around 25% of total bacteria present in feces in *Tcra^−/-^* TAC mice compared to 9% in the WT TAC group (Figure 5f), in line with the enrichment of the “L-tryptophan synthesis” metabolic pathway we observed in Figure 5d. Taken together, *Tcra^−/-^* TAC mice show enrichment of bacteria that produce SCFA- and tryptophan compared to WT TAC mice. The metabolic pathway analysis also suggests that SCFA and/or tryptophan derivatives from bacterial metabolism may contribute to immunomodulation in distant sites from the gut.

### AhR *expression levels in the LV and intestine are downregulated during systolic dysfunction*

**Figure 6.**
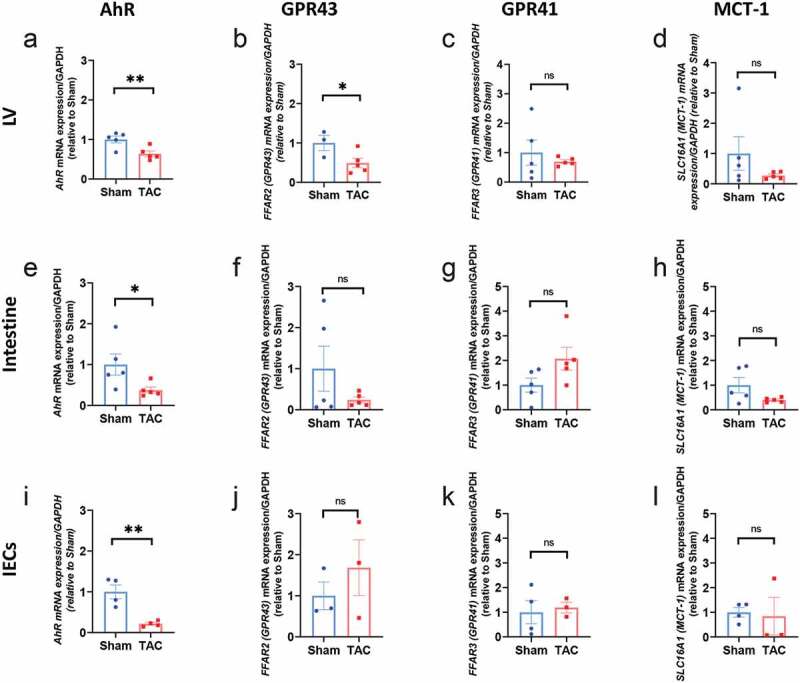
**Reduced *AhR* gene expression in the LV, intestine and IECs in response to cardiac pressure overload induced by TAC** (a, e, i). RNA was isolated from the LV, the intestine, and the IECs 4 weeks post Sham or TAC, and RT-qPCR was performed for the indicated genes *AhR*, SCFA receptors (*FFAR2* and *FFAR3*), and *SLC16A1* (MCT-1, a SCFA transporter). Gene expression levels were measured by in the left ventricle (LV), intestine and intestinal epithelial cells (IECs). Fold changes are shown related to Sham. n = 3–5 mice per group. Each dot represents one mouse. Error bars represent mean ± SEM (Student t-test was used for statistical analysis; *p < .05; **p < .01; ns: no significant)

**Figure 7.**
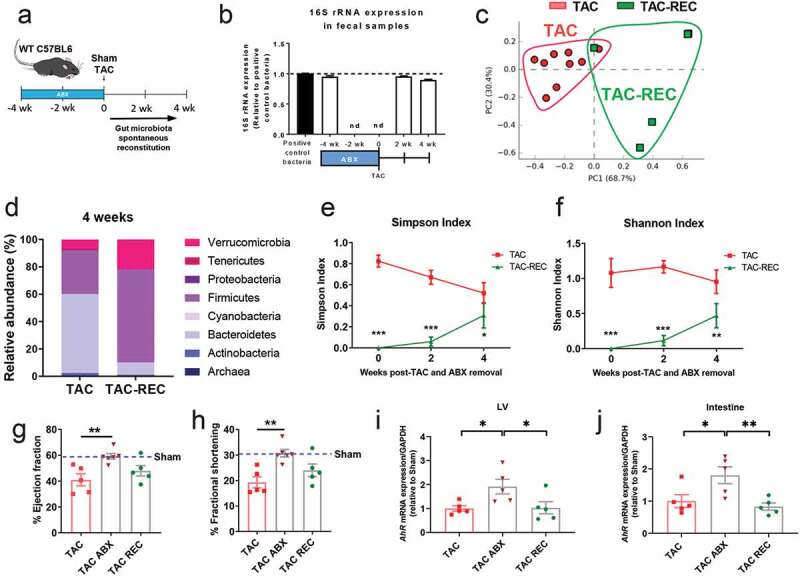
**Spontaneous gut microbiota reconstitution in the onset of TAC results in systolic function comparable to WT TAC, and rescues the decreased expression of *AhR* in the LV and intestine in response to TAC**. (a), Experimental design for gut microbiota spontaneous reconstitution after ABX treatment. (b), 16S rRNA expression measured by qPCR at the indicated times post TAC and ABX removal. Principal component analysis (PCA) clustering of gut microbial populations generated after 16S rRNA sequencing of fecal pellets (c) and relative abundance of main phyla (d) of TAC and TAC-REC mice at 4 weeks post-surgery. (e),(f), estimated microbial alpha diversity indexes (Simpson and Shannon indexes) in TAC mice and TAC REC mice. Ejection fraction (g) and fractional shortening (h) were measured by echocardiography. *Ahr* gene expression was measured by qPCR in the LV (i) and intestine (j). Each point represents one mouse. ANOVA test was used to assess the data. In addition, 2 way ANOVA and Tukey’s multiple comparison test were used for C and D. *p < .05; **p < .01; ***p < .001. wk: week

We next evaluated the gene expression of the main receptors of metabolites derived from the enriched pathways observed in *Tcra^−/-^* TAC vs WT TAC mice, focusing in the intestine, the main site for bacteria metabolism, and in the heart: the expression of GPR43 (*FFAR2* gene) and GPR41 (*FFAR3* gene), receptors for SCFAs, as well as MCT-1, an SCFA transporter; and *AhR*, a receptor for tryptophan derivatives, was determined by qPCR. Of all SCFA-related receptors analyzed, only GPR43 mRNA levels were decreased in the LV due to cardiac pressure overload (Figure 6b), but not in the intestine or in intestinal epithelial cells (IECs) (Figure 6c, 6d, 6f-6h and 6j-6l). However, *AhR* gene expression was significantly reduced in LV, in the intestine and in IECs during TAC (Figure 6a, 6e and 6i). Interestingly, this effect was reverted in mice depleted of microbiota in both LV and intestine (Figure 7i, 7j). Taken these results together, gut dysbiosis induced by cardiac pressure overload prompts changes in *AhR* gene expression in local and distal organs, and may play a role in immunomodulation during TAC.

### *Gut microbiota reconstitution in the onset of TAC results in partial cardiac dysfunction and decreased of* AhR *gene expression*

We next investigated whether spontaneous reconstitution of the gut microbiota in the onset of TAC reversed the preserved systolic function observed when the microbiota is depleted. The gut microbiota was depleted with the ABX cocktail previously described for 4 weeks prior to TAC, and the ABX cocktail was removed at the time of Sham or TAC surgery to allow spontaneous bacterial recolonization of the gut (Figure 7a).

Reconstitution (REC) was effective and we detected bacteria at 2 and 4 weeks after ABX treatment cessation (Figure 7b) by qPCR of 16S rRNA expression in fecal samples. However, 16S sRNA analysis revealed that the microbiota of TAC-REC mice did not match the TAC gut microbiota profile (Figure 7c) in terms of relative abundance of phyla (Figure 7d), and alpha diversity, which was significantly lower in TAC-REC mice overtime (Figure 7e and 7f). Despite an incomplete reconstitution of the microbiota that resembled TAC mice, the progressive acquisition of bacterial populations in the onset of TAC resulted in a decreased percentage of ejection fraction and percentage of fractional shortening that was similar to TAC mice at the 4-week time point (Figure 7g, 7h, Table 2) and lower than TAC ABX mice when these two groups were directly compared (Table 2). Moreover, spontaneous reconstitution of the microbiota in TAC-REC mice resulted in decreased gene expression of *AhR* in both the intestine and in the LV, as compared to TAC ABX mice at 4 weeks (Figure 7i, 7j). Taken together, the spontaneous reconstitution of the microbiota in the onset of TAC results in similar systolic dysfunction and *AhR* gene expression levels as what is observed in TAC mice.

### Lack of T cells and microbes confer protection from TAC and modulate the expression of genes in the tryptophan/AhR pathway

**Figure 8.**
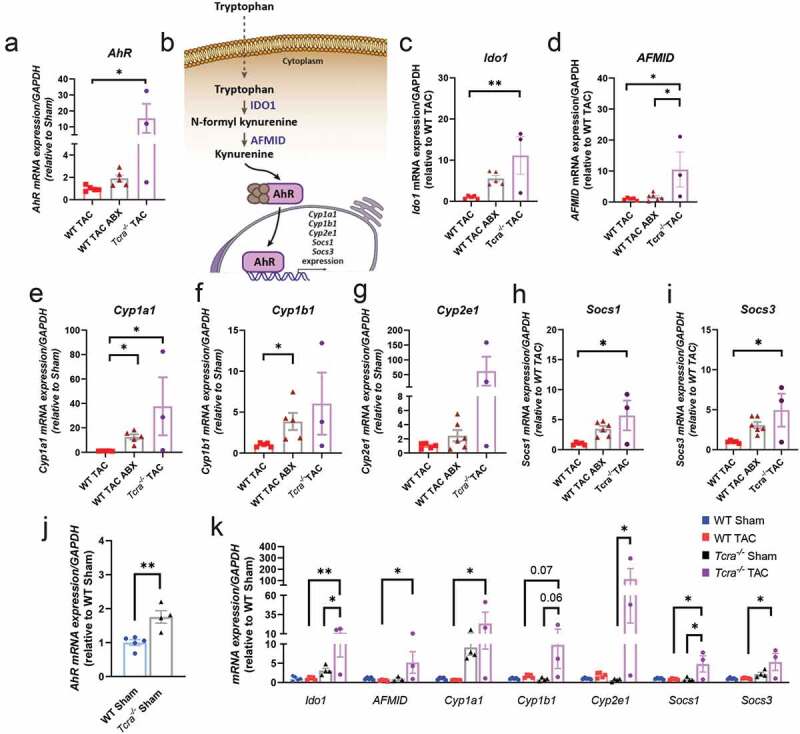
**Absence of gut microbiota or T cells (*Tcra^−/-^*) result in increased gene expression of *AhR*, *AhR* downstream genes, and the enzymes involved in tryptophan catabolism IDO1 and AFMID in response to TAC**. (a), Heart *AhR* gene expression in WT TAC, WT TAC ABX and *Tcra^−/-^* TAC mice. (b), Schematic representation of tryptophan/kynurenine metabolism and AhR signaling pathway. (c-i), Heart gene expression of the following genes was measured by RT-qPCR: genes that encode for metabolic enzymes (c, *Ido1*; d, *AFMID*); and genes that can be transcriptionally regulated by AhR, such as *Cyp1a1* (e), *Cyp1b1* (f), *Cyp2e1* (g), *Socs1* (h) and *Socs3* (i). **J**, Heart *AhR* gene expression at baseline in Sham WT and Sham *Tcra*^*−/-*.^ k, Heart gene expression of the same genes in WT and *Tcra^−/-^* Sham mice (baseline), and in response to TAC. For statistical analysis, we used parametric ANOVA test. Each dot represents one animal. *p < .05; **p < .01

Thus far, we have demonstrated that *AhR* gene expression is decreased in the LV during systolic dysfunction induced by TAC (WT TAC, WT TAC-REC groups), but not in mice depleted of the gut microbiota (WT TAC-ABX mice), which additionally present decreased T cell activation and preserved systolic function. Therefore, we wondered whether *Tcra^−/-^* mice which are also protected for systolic dysfunction, had increased *AhR* expression compared to WT TAC mice. Strikingly, *AhR* mRNA levels were significantly augmented in *Tcra^−/-^* TAC mice compared to WT TAC mice (Figure 8a). We then hypothesized that the tryptophan/AhR pathway (Figure 8b) may play a role in HF development. Because AhR is bound by tryptophan metabolites, and our studies indicated enrichment of tryptophan-producing bacteria in *Tcra^−/-^* mice (Figure 5), we next evaluated the gene expression of two enzymes that degrade tryptophan to secondary metabolites, IDO1 and AFMID, and found that both were increased in *Tcra^−/-^* as compared to WT mice in response to TAC (Figure 8c, 8d). Moreover, we analyzed several genes downstream of AhR signaling that belong to cytochrome P450 families and suppressor of cytokine signaling genes *Socs1* and *Socs3*. The expression levels of *Cyp1a1* and *Cyp1b1* were significantly increased in gut microbiota-depleted TAC mice and *Tcra^−/-^* TAC mice compared with WT TAC mice (Figure 8e, 8f), while *Cyp2e1* remained unchanged (Figure 8g). In addition, *Socs1* (Figure 8h) and *Socs3* (Figure 8i) were also increased in *Tcra^−/-^* mice during TAC, and a trend toward an increase was observed in TAC mice lacking the microbiota. Moreover, we found that the sole deficiency of T cell as at baseline (Sham mice) resulted in a 2 fold increased *AhR* gene expression compared to WT Sham (Figure 8j). *Ido1, Cyp2a* and *Socs1* were also found significantly increased in TAC *Tcra^−/-^* mice, compared to Sham *Tcra^−/-^* mice (Figure 8k). Our results indicate that the absence of T cells, and, to a lesser extent, the absence of microbiota modulate the tryptophan/AhR axis gene expression and may contribute through this pathway to the afforded protection from TAC-induced HF in these conditions.

## DISCUSSION

Our results demonstrate that alterations of the gut microbiota contribute to T cell immune responses in response to cardiac pressure overload induced by TAC, a well-established experimental model of non-ischemic HF. First, we provide evidence that TAC induces gut dysbiosis and characterize the bacteria phyla and genera associated with adverse cardiac remodeling and dysfunction induced by TAC. Second, we demonstrate that such alterations are dependent on T cell presence, since they were not observed in TAC *Tcra^−/-^* mice, which, as we have previously described, are protected from cardiac dysfunction induced by TAC.^19,21^ This, together with the lack of immune cell activation in the gut lamina propria in response to TAC, support that heart T cell immune responses induced by TAC additionally contribute to gut dysbiosis induced by cardiac pressure overload. Third, we demonstrate that sterilizing the gut of microbiota prevents the development of adverse cardiac remodeling and dysfunction through mechanisms that involve decreased T cell activation and leukocyte infiltration in the heart. Lastly, we identify a gene expression program associated with this protection that is characterized by increased expression of host enzymes that metabolize bacterial-produced tryptophan, the AhR and its downstream transcription of AhR target genes in the heart. Our findings in *Tcra^−/-^* Sham and TAC mice further indicate that the regulation of this tryptophan/AhR axis is T cell-dependent.

Gut dysbiosis has been reported in patients with HF from different etiologies.^23,27–31^ The current view is that intestinal congestion in HF patients leads to bacterial leakage and further negatively impact disease progression.^1^ However, experimental models are needed to specifically delineate whether bacteria drive cardiac pathology, or whether it is the other way around. Our studies using a well-controlled experimental model of non-ischemic HF provide evidence that TAC induces significant changes in the microbiota at the phylum level, at a time point (10 weeks) that likely mimics chronic-HF, but additionally show changes earlier on (4 weeks). Interestingly, we do not find signs of gut barrier disruption in intestinal epithelial cells isolated from TAC mice vs Sham mice, suggesting that alterations in the microbiota itself can have distal effects in the heart preceding the intestinal congestion observed in HF,^28,32^ as previously described in other chronic inflammatory models.^11^ It is, however, possible, that given the nature of the TAC model, a sudden increase in cardiac pressure overload may lead to intestinal pressures that result in gut barrier disruption in the compensatory phase post TAC (1 day to 1 week post-TAC), a time point which we have not evaluated in our studies involving intestinal epithelial cells. Our studies further indicate that mice subjected to TAC undergoing progressive adverse cardiac remodeling (at 4 weeks and 10 weeks) have both decreased presence of Firmicutes, a phylum that involves segmented filamentous bacteria, associated with homeostatic gut immune responses,^33^ and increased Bacteroidetes, mostly associated with pro-inflammatory immune responses.^11^ This is in line with our results at the genus level, in which *Lactobacillus*, part of the Firmicutes phylum, is significantly decreased in TAC. Several *Lactobacillus* species are used as probiotics with anti-inflammatory properties that metabolize tryptophan and produce SCFAs, while others, are involved in lactate metabolism and may lead to metabolites with pro-inflammatory activities.^14,34^ While our microbiota analysis does not reach the species identification level, our data showing decreased *Lactobacillus* in TAC at 4 and 10 weeks, when there is significant cardiac inflammation, suggest that cardiac pressure overload over time results in decreased *Lactobacillus* species with anti-inflammatory activity, although this will require further characterization at the species level.

Perhaps our most striking finding is that depletion of microbes, a so-called “gut sterilization,” prevents the development of all hallmarks of adverse cardiac remodeling in response to TAC, including cardiac fibrosis, cardiac hypertrophy, and systolic dysfunction. Unexpectedly, ABX treatment resulted in increased heart rate, compared with control-treated mice. While we do not know the mechanism for this, there could be several possible explanations. Fat composition and weight have been associated with different responses to anesthesia in mice;^35^ however, we think this is unlikely because ABX-treated mice had comparable weights as no ABX-treated mice. It is possible that ABX modulate the anesthesia effect, that sudden changes in microbiota content alter basal heart rate, or that other nonspecific effects of the ABX can have effects on the heart rate response. Future studies will be needed to clarify these mechanisms. We did not observe significant changes in cardiac output among groups. To more accurately determine the ability of TAC and TAC ABX to maintain cardiac output to meet metabolic needs, these values could be analyzed after exercise. Although we did not perform this analysis, we speculate TAC ABX will do better based on the lack of fibrosis and improved systolic function.

Moreover, microbiota depletion resulted in a blunted T cell activation in the mediastinal LNs, and infiltration of T cells in the heart, something we have previously described as being critical for the development of cardiac fibrosis and systolic dysfunction in response to TAC.^19–22^ CD11b^+^ myeloid cells, which precede T cell infiltration in TAC,^36^ were also decreased, suggesting that microbes shape innate and adaptive immune responses in the onset of TAC. Our results are in line with recent studies demonstrating that depletion of the microbiota is protective in hypertension,^16^ and in a model of T cell-induced autoimmunity in mice,^15^ both of which characterized by activated T cell-mediated pathology. In contrast, depleting the microbiota is detrimental in healing post-MI, a model in which the absence of T cells induces the same cardiac rupture phenotype observed with the microbiota depletion.^14^ Thus, all these studies are in line with our findings supporting that microbes facilitate T cell immune responses in cardiac remodeling. Our results demonstrating that *Tcra^−/-^* mice in response to TAC have microbiota profiles that significantly differ from WT TAC mice and resemble healthy mice, also support a critical microbiota/T cell axis in adverse cardiac remodeling. Our studies further reveal that T cell activation in response to TAC is limited to the mediastinal LN, and is not observed in the intestine lamina propria or in the mesenteric LNs that drain the intestine. These results suggest that T cell activation in response to TAC is not imprinted in the gut by commensal *Bacteroides*, something recently described in a spontaneous model of cardiac autoimmunity.^15^ While we do not identify the specific mechanism of T cell activation by bacteria or whether specific bacterial antigenic proteins may mimic cardiac neoantigens, our results provide evidence that gut microbes favor T cell activation at distal sites, rather than in the intestine. The parallelism observed in *Tcra^−/-^* and microbiota depleted mice in response to TAC support that both microbes and T cells are required for the progression of cardiac fibrosis, inflammation, and systolic dysfunction.

Our characterization of the microbiota in *Tcra^−/-^* mice in response to TAC, and the fact that T cell activation in response to TAC in WT mice was not taking place in the gut, where immune cells and microbes co-exist, made us hypothesize that the effects of the microbiota in T cell activation was through metabolites processed by specific bacteria. Our identification of *Lactobacillus* enrichment in *Tcra^−/-^* Sham and TAC mice compared to WT TAC mice, as well as SCFAs and tryptophan metabolic pathways, associated with the protection of adverse cardiac remodeling in response to TAC in *Tcra^−/-^* mice, suggest that these may be exerting cardioprotective effects. Our data in *Tcra^−/-^* Sham mice having increased relative abundance of tryptophan producers, such as genera *Lactobacillus, Clostridium* or *Parabacteroides*. further support that T cells may be involved in controlling several bacterial populations at baseline. This, along with the increased *AhR* gene expression, may contribute to slow down the development of adverse cardiac remodeling with a final result of a better systolic function. Supplementation of SCFAs and *Lactobacillus*-based probiotics have indeed been demonstrated to have cardioprotective effects,^14^ and tryptophan supplementation deemed to be anti-inflammatory in other experimental models of chronic inflammation.^37,38^ Our results indicating that the SCFA receptor GPR43 and the AhR are both decreased in the heart in response to TAC further support a protective role for these metabolites. Deficiency of GPR41, another SCFA receptor, results in spontaneous hypertension,^39^ however TAC did not result in decreased GPR41 expression levels in either the gut or the heart in our studies. Moreover, while GPR43 was only decreased in the heart in response to TAC, AhR was significantly decreased in the heart, the intestine, and specifically in intestinal epithelial cells of TAC mice, suggesting a central multiorgan role. Interestingly, *AhR^−/-^* mice present vascular alterations, cardiomyocyte dysfunction, cardiomegaly and lymphocytic infiltrates,^40^ as well as augmented transforming growth factor (TGF)-β1 and cardiac fibrosis,^41^ and cardiomyocyte-specific deletion of its nuclear translocator, Ahrnt, results in cardiomyopathy.^42^ Our results also indicate that in mice protected from systolic dysfunction in response to TAC by either microbiota depletion, or genetic deficiency of T cells, AhR is not downregulated in response to TAC. The fact that spontaneous reconstitution of the microbiota prevents this effect further supports a cardioprotective role for AhR in the intestine and in the gut. Noteworthy, the genus *Lactobacillus* is a major AhR ligand producer,^26^ and its relative abundance was significantly increased in *Tcra^−/-^* mice, further supporting such cardioprotective role. In the same line of a cardioprotective role of the tryptophan/AhR axis, our results reveal that IDO and AFMID, involved in catabolizing tryptophan and producing the AhR ligand kynurenine, are also increased in *Tcra^−/-^* mice and mice depleted of the microbiota. Interestingly, kynurenine ligation of AhR has been recently proven to be anti-inflammatory in neuroinflammation,^43^ and our studies suggest that it may protect from cardiac inflammation in the TAC setting.

Our studies indicating increased expression of the AhR downstream genes *Cyp1a1, Cyp1b1, Socs1* and *Socs3* in microbiota and T cell-deficient mice, protected from systolic dysfunction, further suggest that the tryptophan/AhR axis activity is required for cardioprotection in response to TAC. These results are in agreement with the well-established pathogenic role of Th1 cells and IL-6 in TAC-induced HF, since SOCS1 and SOCS3 inhibit IFNγ signaling, and IL-6, respectively,^44,45^ and further support an anti-inflammatory role of tryptophan/AhR axis in cardiac inflammation induced by TAC.

Despite some existing limitations in this study that should be noted, we provide, to the best of our knowledge, the first experimental evidence that microbiota depletion protects from systolic dysfunction and adverse cardiac remodeling in response to TAC in a T cell-dependent manner, characterize the microbiota of *Tcra^−/-^* mice in response to TAC, and unveil a possible cardioprotective tryptophan/AhR pathway that is predominantly regulated by T cells, and, to a lesser extent, by the microbiota. Our results suggest that AhR may be a mediator in this protection predominantly conferred by the absence or T cells. It is possible that the absence of microbiota, through decreased T cell activation, regulates *AhR* expression, or that this regulation is independent of T cells and dependent on metabolic cues regulated by the microbiota. Future studies in global or cell-specific tryptophan/AhR pathway gene-deficient mice will be needed to conclude that T cell or microbiota deficiency confers cardioprotection through this pathway, and the specific players in the pathway involved.

One limitation in our studies is that we have limited our metabolic pathway analysis to 16S rRNA sequencing. Metabolomic profiling will determine which tryptophan derivatives trigger AhR activation and in which cells in future studies. Another limitation of all studies involving the microbiota and immune cell activation is that mice purchased from different sources have different microbiota profiles that can determine T cell activation, as previously reported.^33,46^ Nevertheless, mice used in our studies were purchased from Jackson labs and were bred for generations in our animal facilities under controlled temperature, water, diet, and day/night cycle conditions. Slight changes between mouse vendor and facilities may explain some differences with other studies performed in TAC model. For instance, TMAO has been reported to contribute to worsening the pathogenesis of TAC, and elegant studies demonstrate the translational potential of targeting this pathway in patients with HF.^1^ However, choline-associated metabolic pathways related to TMAO were not relatively abundant in TAC mice in a way that was statistically significant in our metabolic pathway analysis. We interpret these differences to be related to the animal facilities’ conditions or mouse sources. This limitation does not detract from our internally controlled findings demonstrating the gut dysbiosis induced by TAC, and from identifying bacterial genera associated with cardioprotection in *Tcra^−/-^* mice. Lastly, while our results reveal new pathways of cardioprotection afforded by either the lack of microbiota or the lack of T cells, whether the lack of T cell immunity shapes the microbiota toward cardioprotection via maintenance of AhR programs, or whether specific bacteria can prevent T cell activation and adverse cardiac remodeling through AhR signaling, still requires further investigation and is an ongoing area of investigation in our group.

In conclusion, our study supports the relevance of gut dysbiosis induced by TAC in T cell immune responses and provide novel mechanistic insight to ameliorate cardiac dysfunction by modulating T cell immune responses through microbiota-produced metabolites, and immunomodulation of AhR signaling.

## MATERIAL AND METHODS

The data, analytical methods, and study materials for the purposes of reproducing the results or replicating procedures can be made available on request to the corresponding author.

### Mice

WT C57BL/6 and *Tcra^−/-^* male mice of 8–10 weeks of age were purchased from Jackson Laboratory, USA, and maintained under pathogen-free conditions in the Tufts University animal facilities for several generations. All mice were single housed in different cages to avoid homogenous microbiota due to coprophagy, never mixing mice from different experimental conditions. Animals had access to food and water *ad libitum* and were maintained in a 12 h light/dark cycle. All protocols and procedures conformed to the animal welfare regulations of Tufts University and were approved by the Tufts University Institutional Animal Care and Use Committee. All experiments involving animals were conducted in accordance with the *Guide for the Use and Care of Laboratory Animals*.

### Mouse Model of Transverse Aortic Constriction

Transverse aortic constriction (TAC) was used to induce left ventricle (LV) pressure overload–induced HF as previously described.^47–49^ Sham mice were subjected to the same procedure but without constriction. WT and *Tcra*^−/-^ mice were harvested at 4- and 10-weeks post-TAC.

### ABX Treatment

Drinking water supplemented with a well-established broad-spectrum ABX cocktail,^50–52^ that contained ampicillin (1 g/L, Sigma-Aldrich), metronidazole (1 g/L, Sigma-Aldrich), neomycin sulfate (1 g/L, Sigma-Aldrich) and vancomycin (0.5 g/L, Sigma-Aldrich) was provided for depleting gut microbiota. Moreover, sucrose 0.2% was added to make ABX cocktail more palatable. For the depletion experiment, ABX treatment was started 1 week before TAC surgery and was terminated 4 weeks post TAC. For the reconstitution experiment, mice received ABX cocktail 4 weeks prior to Sham/TAC surgery and ABX treatment was removed the day of surgery, leaving mice to a spontaneous gut microbiota reconstitution.

### In vivo *transthoracic echocardiography*

*In vivo* echocardiography was evaluated in barely sedated mice as described.^49^ M-mode and two-dimensional images were obtained from the short-axis view, as previously described.^48^ Blinded analyses were performed.

### In vivo *hemodynamics*

At the end of each experiment, invasive hemodynamics were recorded using a LV conductance catheter as described,^49^ and data were digitized and analyzed using EMKA 2.1.10 software.

### Flow cytometry characterization of immune cells from lymph nodes and lamina propria

Mediastinal and mesenteric LNs were harvested to study T cell activation and evaluate the expression of IFNγ and interleukin (IL)-17A by flow cytometry analysis. In addition, lamina propria immune cell populations were analyzed by flow cytometry.

Briefly, lymph nodes were broken up and resuspended in FACS buffer (PBS + 2% fetal bovine serum (FBS)). For surface staining, isolated cells were incubated with anti-CD16/CD32 (for blocking nonspecific binding of immunoglobulin to the Fc receptors, clone 93), anti-CD3 (clone 145–2 C11), anti-CD4 (clone GK1.5), anti-CD11b (clone M1/70), anti-CD11c (clone N418), anti-CD44 (clone IM7), anti-CD45.2 (clone 104), anti-B220/CD45R (clone RA3-6B2) or anti-CD62L (clone MEL-14) (all antibodies from BioLegend). For intracellular staining, cells were pre-incubated with a cocktail that contained brefeldin, monensin, PMA, and ionomycin, for 4h at 37°C. A fix/perm kit (BioLegend) was used, and cells were stained with anti-IFNγ (clone XMG1.2), anti-IL-17A (clone TC11-18 H10.1).

For isolation of intestinal epithelial cells (IECs) and immune cells from the lamina propria, we used the protocol previously described by Couter and Surana,^53^ with minimal changes. Concisely, we digested the tissue with RPMI supplemented with FBS, DTT (Sigma-Aldrich) and EDTA (Gibco), and separated IECs and lamina propria. Then, we processed the remaining tissue (lamina propria) with collagenase II (Gibco) and filtered it to obtain immune cell populations. Cells were stained as described previoulsy. At least 100,000 events were acquired in each experiment on an LSRII flow cytometer (Becton Dickinson) and analyzed using FlowJo software.

### Histological analysis

Cardiac tissue was fixed in 10% formalin (Sigma), embedded in paraffin and cut into 5 μm sections. Hematoxylin and eosin (H&E) or picrosirius red staining were performed as previously described.^19^ Cardiomyocyte cross-sectional area was quantified by tracing the outline of at least 20 myocytes per section.^19,54^ Collagen deposition was quantified using ImageJ software (NIH; Bethesda, MD).

For immunohistochemistry, frozen sections were fixed in acetone, washed, and incubated with 10% normal goat serum for blocking nonspecific binding for 1 h at room temperature. Then, sections were incubated with an avidin/biotin blocking kit (Vector Laboratories) in the dark at room temperature during 15 min each. After blocking, sections were incubated with rat anti-mouse primary antibodies overnight at 4ºC against CD4 (1:500 dilution; BioLegend), followed by goat anti-rat biotinylated secondary antibody (1:1,000; Vector Laboratories). For the next step, sections were incubated with ABC-Elite-Standard Kit for Peroxidase (PK6100, Vector laboratories), and then with aminoethyl carbazole (AEC) solution. Hematoxylin was used as a nuclear counterstaining. Sections were mounted with Aqua-mount (Lerner laboratories) and covered with a coverslip.

### Sample collection, 16S rRNA sequencing and data processing

Fecal samples were collected from each mouse directly into individual sterile tubes and stored at −80°C for rRNA extraction. rRNA isolation was performed using a phenol-chloroform extraction (Trizol, Invitrogen) and ethanol precipitation, and was purified (AllPrep DNA/RNA Mini Kit, Qiagen). Library preparation, sequencing, and QIIME analysis were performed by the Tufts University Core Facility Genomics Core. For each sample, an amplicon library of the 16S rRNA gene was constructed by performing PCR using primers flanking the variable region 4 (V4) (note: the primer pairs are actually on the constant region of 16S RNA), followed by a nested PCR to introduce Illumina adaptors. These 16S rRNA amplicon libraries were then mixed in equal molar concentration and sequenced with an Illumina MiSeq using MiSeq V2 chemistry and paired-end 250 format. The subsequent raw data were transformed to fastq format using bcl2fastq from Illumina. Each sequence was assigned to corresponding operational taxonomic units (OTUs) based on a minimum 97% identity (closed OTU picking), and the frequency of OTUs from all samples was summarized at multiple taxonomy levels.

*Changes in relative abundance, principal component analysis (PCA), diversity indexes and volcano plots*: Normalized OTU tables for fecal samples, with accompanying metadata of group (Sham, TAC, TAC reconstituted (REC) mice), treatment (ABX/no ABX), strain (WT, *Tcra^−/-^*) and timepoint (0, 2, 4 and 10 weeks post-surgery), were imported into the open-source software STAMP v2.1.3^55^ for statistical analysis. Alpha diversity (Simpson and Shannon indexes) was calculated importing OTUs genera frequency from all samples into the free software Past3.2 (https://folk.uio.no/ohammer/past/). Volcano plots were done with GraphPad Prism 8 software.

### Real-Time Quantitative PCR for quantification of tissue mRNA

Total RNA was extracted from the LV, proximal colon, and intestinal epithelial cells (IECs) with QIAzol (Qiagen) and converted to cDNA with the ThermoScript RT-PCR system (Invitrogen). Then, it was amplified with SYBR green PCR mix (Applied Biosystems). Samples were quantified in triplicates using 40 cycles performed at 94°C for 30 sec, 60°C for 45 sec and 72°C for 45 sec using a QuantStudio 6 Flex (Applied Biosystems) with its respective software. The list of primers used in this study is in Table1.

### Endotoxin ELISA

Endotoxin concentrations were determined with Pierce LAL Chromogenic Endotoxin Quantitation Kit (Thermo Fisher Scientific) following manufacturer’s instructions. Serum from mice intraperitoneally injected with LPS (E. coli, Sigma), 15 µg LPS/g mouse for 4 h, was used as a positive control.

### Statistical Analysis

All results are presented as mean ± SEM. GraphPad Prism 8 software was used for statistical analysis, applying parametric Student’s t-test and ANOVA, or non-parametric Kruskal-Wallis or Mann-Whitney U tests. Multiple group comparisons were performed by one- or two-way ANOVA and Bonferroni posttest, unless otherwise indicated. For statistical analysis and dynamic identification of microbiome differences by relative abundance between the groups, STAMP v2.1.3 software was used. A Welch´s t-test for unequal variances was used to assign *p*-values to the OTUs when comparisons between groups were done. Values of *p < .05; **p < .01 and ***p < .001 were considered statistically significant.
